# Sequence Polymorphism and Intrinsic Structural Disorder as Related to Pathobiological Performance of the *Helicobacter pylori* CagA Oncoprotein

**DOI:** 10.3390/toxins9040136

**Published:** 2017-04-13

**Authors:** Hiroko Nishikawa, Masanori Hatakeyama

**Affiliations:** 1 Division of Microbiology, Graduate School of Medicine, The University of Tokyo, 7-3-1 Hongo, Bunkyo-ku, Tokyo 113-0033, Japan; hiroco007@m.u-tokyo.ac.jp; 2 CREST, Japan Science and Technology Agency, Saitama 332-0012, Japan; 3 Max Planck-The University of Tokyo Center for Integrative Inflammology, Tokyo 113-0033, Japan

**Keywords:** *Helicobacter pylori*, CagA oncoprotein, gastric carcinoma, CagA polymorphism, EPIYA motif, CM motif, intrinsically disordered region, scaffold/hub protein

## Abstract

CagA, an oncogenic virulence factor produced by *Helicobacter pylori*, is causally associated with the development of gastrointestinal diseases such as chronic gastritis, peptic ulcers, and gastric cancer. Upon delivery into gastric epithelial cells via bacterial type IV secretion, CagA interacts with a number of host proteins through the intrinsically disordered C-terminal tail, which contains two repeatable protein-binding motifs, the Glu-Pro-Ile-Tyr-Ala (EPIYA) motif and the CagA multimerization (CM) motif. The EPIYA motif, upon phosphorylation by host kinases, binds and deregulates Src homology 2 domain-containing protein tyrosine phosphatase 2 (SHP2), a *bona fide* oncoprotein, inducing pro-oncogenic mitogenic signaling and abnormal cell morphology. Through the CM motif, CagA inhibits the kinase activity of polarity regulator partitioning-defective 1b (PAR1b), causing junctional and polarity defects while inducing actin cytoskeletal rearrangements. The magnitude of the pathobiological action of individual CagA has been linked to the tandem repeat polymorphisms of these two binding motifs, yet the molecular mechanisms by which they affect disease outcome remain unclear. Recent studies using quantitative techniques have provided new insights into how the sequence polymorphisms in the structurally disordered C-terminal region determine the degree of pro-oncogenic action of CagA in the gastric epithelium.

## 1. Introduction

Chronic infection with *H. pylori* is associated with major upper gastrointestinal diseases such as atrophic gastritis, peptic ulcerations, and gastric cancer [1,2,3]. With 951,000 new cases and 723,000 deaths in 2012, gastric cancer is the third leading cause of cancer-related deaths worldwide and accounts for a significant portion of the global cancer burden [4]. East Asian countries including China, Japan and South Korea have the highest incidences of gastric cancer, accounting for more than half of all new cases of gastric cancer worldwide each year. It is notable that 98% of all gastric cancers in Japan are causally associated with *H. pylori* infection, highlighting the importance of *H. pylori* eradication for the prevention of gastric cancer in East Asia [5]. Evidence from both epidemiological and experimental studies points to a strong link between the regional propensity in the incidence of gastric cancer and the C-terminal sequence polymorphisms of the *H. pylori* effector protein, cytotoxin-associated gene A (CagA) [6,7].

*H. pylori* CagA, the only bacterial oncoprotein identified to date, has a molecular weight that ranges between 120 and 145 kDa due to its C-terminal polymorphisms arising from variable repeat expansion [8,9]. It is encoded by the *cagA* gene that is located in the *cag* pathogenicity island (*cag* PAI), a 40-kb DNA fragment that is speculated to have been acquired through horizontal transfer, although its origin remains unknown [10,11]. The *cag* PAI contains up to 30 genes other than *cagA*, many of which encode proteins that make up the type IV secretion system, a molecular syringe that delivers the bacterial CagA protein into the host cell. Almost all East Asian strains of *H. pylori* contain the *cag* PAI and are thus *cagA*-positive, while only about 60% of Western strains contain the *cag* PAI. *H. pylori cagA*-positive strains are known to be more virulent, causing more severe forms of gastric mucosal inflammation that lead to the development of peptic ulceration or gastric cancer [12,13].

Structural biology studies using X-ray crystallography have revealed that the N-terminal 70% of the full-length CagA protein has a unique solid structure that does not have any homology with other known proteins [14,15]. The rest (C-terminal 30%), as an isolated domain, has been shown by nuclear magnetic resonance (NMR) and circular dichroism (CD) spectroscopy to be “intrinsically disordered”, a term used in protein chemistry to describe flexible regions that lack stable secondary and tertiary structures under physiological conditions and thus cannot be crystallized [14,16]. As evidence accumulates that intrinsically disordered proteins (IDPs) or regions of proteins (IDRs) are extensively utilized in both physiological and pathological signaling/regulatory pathways [17,18,19,20], much attention has recently been focused on the biological properties of IDPs/IDRs. Conformational study of the C-terminal region in the context of the full-length CagA protein has been hampered by failure of crystallization of full-length CagA, most probably due to the intrinsically disordered and thereby flexible nature of the C-terminal region. Also, the molecular weight of full-length CagA protein (120~145 kDa) is beyond the limits of conventional NMR analysis. Nevertheless, CD spectroscopy analysis of full-length CagA has suggested that the C-terminal CagA region is intrinsically disordered and thus flexible, making it possible to create a lariat by looping back onto the structured N-terminal CagA region [14].

**Figure 1:**
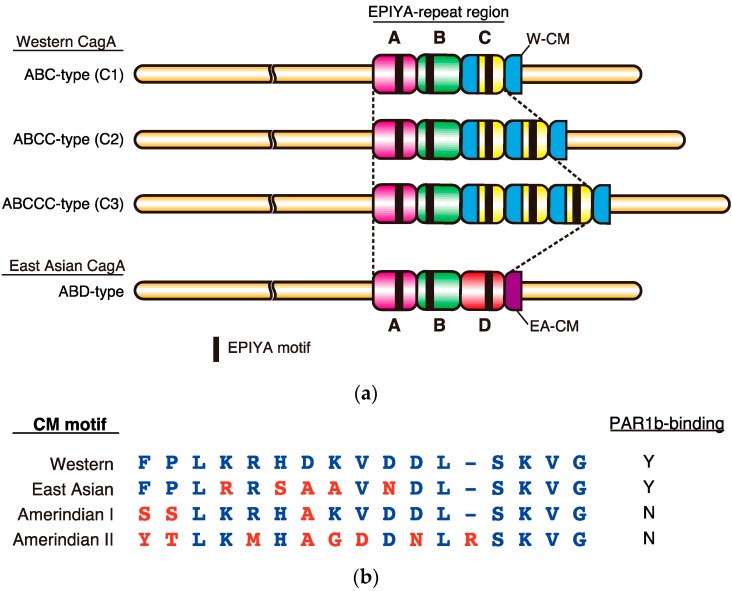
Polymorphisms in the C-terminal tail of *H. pylori* CagA. (**a**) The C-terminus of Western CagA is organized into segmental repeats characterized by EPIYA-A, EPIYA-B and typically 1–3 repeats of EPIYA-C segments, each containing a single EPIYA motif. Note that Western CagA always carries an extra copy of the Western CM motif (W-CM) flanking the distal end of the EPIYA-repeat region. In contrast, the highly oncogenic East Asian CagA usually contains EPIYA-A, EPIYA-B and a single copy of the EPIYA-D segment. Since EPIYA-D segment does not contain a CM motif, East Asian CagA typically carries only one East Asian CM motif (EA-CM), which flanks the EPIYA-repeat region at the distal end; (**b**) Sequence alignment of regional CM variants and their ability to bind PAR1b. Western, East Asian, Amerindian I, and Amerindian II CM motifs were obtained from strains NCTC11637, F32, Shi257, Shi193, respectively. Y; Yes. N; No.

CagA acts as a pathogenic scaffold/hub protein by interacting with a number of host proteins through the intrinsically disordered and thereby flexible C-terminal tail, which contains two types of repeat motifs: the 5 amino-acid residue Glu-Pro-Ile-Tyr-Ala (EPIYA) motif and the 16 amino-acid residue CagA multimerization (CM) motif (Figure 1a,b) [21,22,23]. The EPIYA motif sequence fits the substrate-recognition sequences for Src family kinases (SFKs) and c-Abl, which are DEEIY(G/E)EFF and AXVIYAAPF, respectively, and indeed are targeted by these kinases [24]. Upon delivery into host cells via the type IV secretion system, a cluster of basic amino acid residues in Domain II of the CagA molecule called the “basic patch” tethers CagA to the inner leaflet of the plasma membrane by interacting with phosphatidylserine (PS), a plasma membrane phospholipid [14,25]. Interaction of the basic patch with PS is likely to orientate the C-terminal lariat loop towards the cytoplasm, making it easily accessible for SFKs and c-Abl to target the tyrosine residues in the EPIYA motifs. Tyrosine phosphorylation is essential for CagA to interact with Src homology 2 (SH2) domain-containing proteins such as the pro-oncogenic protein tyrosine phosphatase SHP2, C-terminal Src kinase (Csk), and the adaptor protein Crk [26,27,28]. The CM motif on the other hand, binds to the polarity-regulating serine/threonine kinase partitioning-defective 1 (PAR1), also denoted as microtubule affinity-regulating kinase (MARK) in mammals, independently of phosphorylation [29]. The crystal structure of the complex between the C-terminal fragment of CagA (885–1005 aa of *H. pylori* 26695 CagA) and PAR1 has shown that the CM motif mimics host substrates that bind to the kinase catalytic site of PAR1 [30]. In the crystals, the majority of the C-terminal fragment was disordered and only 14 amino acids of the CM motif bound to the active site were visible, although they did not form any secondary structures. Therefore, at least some portions of the C-terminal tail remain structurally disordered even when they are bound by their targets.

Aberrant pro-oncogenic signals elicited by deregulated SHP2 via the EPIYA motif, together with destruction of the gastric epithelium caused by CM-mediated PAR1 inhibition are two major pathobiochemical processes that cooperatively contribute to *H. pylori* CagA-induced gastric oncogenesis. In this review, we will focus on the sequence polymorphisms of CagA with respect to SHP2 and PAR1 binding. We will highlight recent biochemical and cell biological findings and discuss how the polymorphisms located in the flexible C-terminal tail of CagA quantitatively influence its virulence and affect the outcome of gastrointestinal diseases.

## 2. Sequence Polymorphism in the EPIYA Motif-Containing Region of CagA

The oncogenic potential of CagA is associated with its geographical subtype, which can be classified according to sequences flanking the EPIYA motifs largely into either East Asian CagA, found exclusively in East Asian countries, or Western CagA, which is found in most other parts of the world. The C-terminal EPIYA motif-containing region of CagA is composed of “EPIYA segments”, which are 20–50 amino acids in length (Figure 1a). Four types of EPIYA segments, EPIYA-A, -B, -C, and -D, each of which contains a single EPIYA motif, have been defined on the basis of the context of the sequence spanning each of the EPIYA motifs [31]. Upon tyrosine phosphorylation, the EPIYA motifs in the EPIYA-A and EPIYA-B segments serve as binding sites for Csk, while those in the EPIYA-C or EPIYA-D segments serve as specific binding sites for SHP2 [26,27]. The Western CagA subtype contains EPIYA-A, -B and typically one to three copies (although it can contain up to six, due to repeat expansion) of the 34 amino-acid EPIYA-C segments. The East Asian CagA subtype contains EPIYA-A, EPIYA-B, and usually a single copy of the EPIYA-D segment. In Western CagA, one CM motif is present per EPIYA-C segment. In addition, there exists another CM motif distal to the last repeat of the EPIYA-C segment. The 48-bp *CM* sequences are long enough to serve as homologous recombination donor/acceptor sites and are critical in creating the variable repeat number of EPIYA-C segments in the C-terminal repeat region [32]. In contrast, East Asian CagA rarely duplicates the EPIYA-D segment by homologous recombination because it lacks a *CM* sequence within the EPIYA-D segment, carrying only a single *CM* sequence immediately downstream of the EPIYA-D segment. Thus, East Asian CagA has adopted another strategy for augmenting binding affinity to SHP2 and PAR1: amino-acid substitution within the EPIYA-D segment and the CM motif. The sequence polymorphisms in the EPIYA motif-containing region thereby create differential binding activities of CagA to SHP2 and PAR1, which influence the virulence of individual CagA as discussed below.

## 3. Role of EPIYA Polymorphism in SHP2 Binding

SHP2, encoded by the gene *PTPN11*, is a ubiquitously expressed non-receptor-type protein tyrosine phosphatase that is conserved throughout metazoans [33]. It has diverse physiological roles in cellular proliferation, cell morphology, cell motility, development, and differentiation. In its N-terminus, SHP2 contains two tandem SH2-domains, N-SH2 and C-SH2, followed by a protein tyrosine phosphatase (PTP) domain. N-SH2 is a conformational switch whereby a loop in the N-SH2 domain occludes the catalytic center of the PTP domain through intramolecular interaction allowing for only basal phosphatase activity [34]. Stimulation of cells with growth factors or cytokines creates phosphotyrosine residues on receptor protein kinases and/or adaptor proteins such as Gab proteins, which serve as specific recognition sites by the SHP2 SH2 domains. Once bound to these target phosphotyrosine residues, N-SH2 undergoes a conformational change that releases the PTP domain from autoinhibition, rendering it enzymatically active. SHP2 activity is required for full activation of the ERK MAPK signaling pathway, which conveys a potent mitogenic signal [35,36]. The importance of the SH2 domain in regulation of SHP2 activity is supported by the fact that many diseases have been associated with mutations that disrupt the intramolecular N-SH2/PTP interaction in SHP2 [37]. For example, germline mutations have been found in ~50% of patients with Noonan syndrome, a developmental disorder that is occasionally accompanied by juvenile myelomonocytic leukemia (JMML) [38,39]. Likewise, somatic mutations in *PTPN11* that affect the SH2-PTP interface are found in patients with sporadic JMML [40,41].

The tyrosine-phosphorylated EPIYA-C or EPIYA-D segment of CagA, which mimics endogenous mammalian targets of the SHP2 SH2 domains, may release the PTP domain from SH2 autoinhibition, thereby eliciting aberrant activation of the pro-oncogenic ERK MAPK signaling pathway in addition to inducing an extremely elongated cellular morphology in cultured cells known as the “hummingbird phenotype” [26,34]. Furthermore, the importance of the CagA EPIYA motif in promoting malignant transformation has been demonstrated in vivo by the observation that transgenic mice systemically expressing CagA developed gastrointestinal adenocarcinomas and hematological malignancies in an EPIYA-dependent manner [42,43].

**Figure 2:**
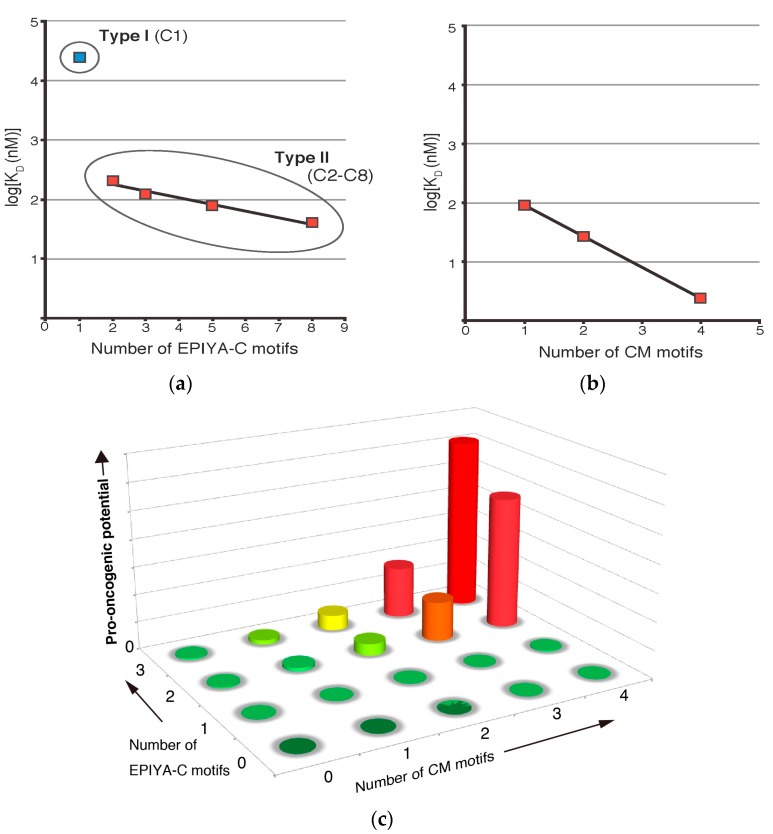
Quantitative analysis of binding affinities between Western CagA, containing varying numbers of EPIYA-C segments, and its targets SHP2 and PAR1b. (**a**) Based on their binding affinities to SHP2, Western CagA species are classified into two groups. Type I CagA refers to low-affinity CagA, which contains only a single copy of EPIYA-C motif. Type II CagA refers to high-affinity CagA species, which contain variably duplicated EPIYA-C repeats (Adapted and modified from [46]); (**b**) Increase in the number of CM motifs per CagA molecule exponentially enhances PAR1b-binding activity (Adapted and modified from [47]); (**c**) A conceptual graph depicting how the increase in the copy numbers of EPIYA-C and CM motifs synergistically contribute to the pro-oncogenic potential of Western CagA.

As stated above, Western CagA variants can possess up to six copies of EPIYA-C, although the frequencies of the variants decrease sharply as the number of EPIYA-C segments increases [32]. The majority (60–70%) of Western CagA contains just one EPIYA-C segment (C1), 20–30% of Western CagA contains two EPIYA-C segments (C2), and less than 5% contains three EPIYA-C segments (C3) (Figure 1a) [44,45]. Quantitative analysis of binding affinity between a recombinant fragment of the two tandem SH2 domains of SHP2 and tyrosine-phosphorylated recombinant Western CagA proteins with one, two, three, five, and eight copies of EPIYA-C segment by surface plasmon resonance (SPR) gave apparent dissociation constants (K_D_) of 24.1 µM, 208 nM, 123 nM, 77.8 nM, and 40.1 nM, respectively [46]. Since stronger bindings give lower K_D_ values, the results clearly indicated that the SHP2-binding affinity increased as the number of EPIYA-C segments increased. However, the augmentation of binding affinity was not linear, with a “boost” observed between one and two copies of EPIYA-C, which showed a 100-fold increase, whereas further augmentation of binding affinity from two to eight copies of EPIYA-C was only 50-fold. Plotting the values on a semi-log scale showed that CagA (C2, C3, C5, C8) can be fitted to a linear regression line but that CagA (C1) was an anomaly and did not fit the line (Figure 2a). This marked difference in SHP2 binding allowed classification of Western CagA into two groups: low affinity CagA (C1) as “Type I” and higher affinity CagA (C2–C8) variants as “Type II”. Since the concentration of endogenous SHP2 in human gastric epithelial cells was estimated to be approximately 100 nM, the K_D_ value of CagA (C1)/SHP2 interaction, which showed 24 µM, would be too weak to induce any significant complex formation. On the other hand, CagA (C2) and CagA (C3) with K_D_ values of 208 nM and 123 nM, respectively, would be able to elicit pathobiologically relevant interaction with SHP2. It is worth mentioning that CagA (C5) is very rare and CagA (C8) has not been reported in clinical isolates to date. This may simply be a case where the cost of making extra EPIYA segments does not meet the benefit, i.e., the increase in binding affinity, as CagA (C2) and CagA (C3) are sufficient to deregulate SHP2.

The “boost” in the SHP2-binding affinity observed between Type I Western CagA and Type II Western CagA is most likely due to the avidity effect via the two tandem SH2 domains of SHP2. When there is only one EPIYA-C segment, the CagA molecule can bind to only one of the tandem domains, generating very weak binding. However, when there is more than one EPIYA-C segment, both SH2 domains can simultaneously engage in binding with the same CagA molecule, generating enhanced binding through the avidity effect. In accordance with the results of in vitro mixing experiments, gastric epithelial cells transiently expressing Type II Western CagA showed more enhanced binding to endogenous SHP2 than did Type I Western CagA. Cells expressing Type II Western CagA also exhibited more enhanced migration and invasive phenotypes on collagen matrix gel than did those expressing Type I Western CagA [46]. These results collectively indicated that EPIYA-C segments of two repeats or more can reach the threshold required to evoke pathobiological effects on host cells.

The EPIYA-D segment, which is specific to East Asian CagA, has been shown to bind to SHP2 more strongly and thus has been linked more closely to oncogenesis than the Western CagA-specific EPIYA-C segment [31]. Also, it has been shown that the residue positioned at +5 from the phosphotyrosine residue in the EPIYA segments, which is phenylalanine in the EPIYA-D segment and aspartic acid in the EPIYA-C segment, is critical for the enhanced SHP2 binding of East Asian CagA. An outstanding question is how the EPIYA-D segment can undergo such an enhanced SHP2-binding despite the presence of only a single EPIYA segment that can bind to SHP2. Further structural studies will be required to address this question in the future.

## 4. Role of CM Polymorphism in PAR1 Binding

The polarity-regulating kinase PAR1 is a serine/threonine kinase conserved from yeast to humans. The gene encoding PAR1 was originally identified in a screening of genes responsible for defective cleavage patterns in the early embryo of *C. elegans* [48]. The mammalian proteome contains four PAR1 orthologues, named PAR1a-d. They are also known as MARK1-4 as they phosphorylate microtubule-associated proteins (MAPs) such as MAP2, 4 and tau, releasing them from microtubules to destabilize the microtubule cytoskeletal system [49,50]. Among the four isoforms, PAR1b is the primary isoform expressed in human epithelial cells. PAR1b localizes to the basolateral inner leaflet of the plasma membrane of polarized epithelial cells and plays crucial roles in both establishment and maintenance of apical-basal polarity [51,52,53]. Consistent with this, inhibition of PAR1b by CagA results in loss of cell polarity, disrupts tight junctions, and induces abnormal cell morphology that resembles epithelial-mesenchymal transition [29,54,55]. PAR1b also suppresses formation of stress fibers, which are contractile bundles of actomyosin that play important roles in cell adhesion and morphogenesis, through phosphorylation-dependent inhibition of a RhoA-specific GEF, GEF-H1 [56].

Crystal structure analysis revealed that the 16 amino-acid CM motif sequence inhibits the kinase activity of PAR1b by directly binding to the active site of the PAR1b catalytic domain, mimicking host substrates [30]. In Western CagA, each EPIYA-C segment contains a single CM motif and there is an extra copy of the CM motif immediately distal to the last repeat of the EPIYA-C segment (Figure 1a). Accordingly, there is always one additional CM motif compared to the number of EPIYA-C segments [23]. In contrast, East Asian CagA possesses a single CM motif, termed the East Asian CM motif as it differs from the Western CM motif by 5 amino-acid residues (Figure 1b). In addition to Western and East Asian CM motifs, new variants have been identified from other parts of the world such as the Amerindian I and II CM motifs, discovered in *H. pylori* CagA isolated from natives of the Peruvian Amazon. However, these CM motifs have more or less lost their ability to bind to PAR1b [57,58,59,60]. In such isolated and small communities, it might be advantageous for *H. pylori* to attenuate its virulence, otherwise it would have a detrimental effect on such a segregated minority population [47].

Quantitative analysis of binding affinity using a saturation binding assay revealed that the K_D_ values of PAR1b and Western CagA with one, two, and four CM motifs were 90.3 nM, 26.6 nM, and 2.43 nM, respectively [47]. These values could be fitted to a linear regression line on a semi-log scale and indicated that an increase in the number of CM motifs per CagA molecule augmented binding affinity to PAR1b in an exponential manner, analogous to the relationship between Type II Western CagA and SHP2 (Figure 2b). Interestingly, East Asian CagA containing a single East Asian CM motif exhibited PAR1b-binding affinity that was comparable to that of Western CagA carrying two Western CM motifs. Considering that these two variants dominate the world, such a level of PAR1b-binding activity may be required and sufficient for successful colonization of the stomach by *H. pylori*.

Gastric epithelial cells transiently expressing CM variants of CagA exhibited stress fiber formation that was concordant with the dissociation constants determined [47]. Furthermore, Madin-Darby canine kidney (MDCK) cell monolayers displayed CM copy number-dependent disruption of the tight junction. These results suggested that individuals infected with *H. pylori* expressing CagA with larger numbers of CM motifs would be at higher risk for severe mucosal damage of the stomach, which would render them more susceptible to oncogenic insults. Taken together, the number of EPIYA-C motifs and the number of CM motifs are two quantitative determinants for the oncogenic potential of individual Western CagA species (Figure 2c).

## 5. Intrinsic Structural Disorder—A Critical Structural Feature for Bacterial Effectors

Like *H. pylori* CagA, many bacterial effectors are thought to contain IDRs, which are critical for their function and virulence [8,61]. Features that make IDRs suitable for use in effector proteins are: (1) flexibility that facilitates interaction with multiple proteins, (2) accessibility to post-translational modification target sequences by modification enzymes, (3) rapid evolution of repeat motifs to confer augmentation of target binding or acquisition of new functions, and (4) cost-effectiveness and compactness. Conventional methods for studying protein structure have facilitated analysis of well-structured proteins, leading to a misconception that a 3D structure is prerequisite for protein function [62]. Although IDPs or proteins containing IDRs account for a significant proportion of the proteome, they were largely ignored and deemed to be non-functional. Only recently, with advancements in genetic engineering, important biological functions are increasingly being mapped to IDRs and we are beginning to appreciate their potential.

The prevalence of IDRs (of over 40 residues) has been predicted to be high (35–51%) in the eukaryotic proteome, while simpler organisms such as prokaryotes have low prevalences of IDR (6–33%) [63]. In humans, enrichment for IDRs has been predicted particularly for cell-signaling and cytoskeletal proteins [64]. Therefore, the high prevalence of IDRs in eukaryotes may be attributed to their complex cell-signaling pathways and cellular structures compared with those of prokaryotes. Many of the human proteins that function in cell-signaling and the cytoskeleton are scaffold proteins. IDRs are suitable as scaffolds because they can bring together multiple proteins, often via post-translational modifications such as phosphorylation, to regulate signal transduction or to promote assembly of protein complexes. Post-translationally modified targets located in flexible IDRs allow them to easily access and fit into the catalytic cleft of modification enzymes. Furthermore, IDRs are economical and less bulky as they expose more surface area per residue, allowing for more binding sites than well-structured regions of the same length [65]. Moreover, IDRs allow rapid evolution of functional domains through repeat expansion as many IDRs contain tandem repeats [66]. Expansion of conserved repeats can generate multivalent bindings, while mutations in the repeats can diversify binding partners and create new functions.

Despite the low abundance of IDRs in prokaryotic proteomes, *H. pylori* CagA and other bacterial effectors are rich in IDRs [8,61]. For example, there is a class of bacterial effectors, which includes *H. pylori* CagA that contains repeatable EPIYA-like tyrosine-phosphorylation motifs and are thereby termed “EPIYA effectors” [67]. These bacterial proteins have no obvious homology to each other apart from the EPIYA-like motifs themselves, which may perturb host-signaling pathways by interacting promiscuously with SH2 domain-containing host proteins. Therefore, the EPIYA-like motifs may have occurred independently in these organisms and have undergone convergent evolution where an advantage in survival during host-pathogen interaction led to the selection of the EPIYA-like motif. Despite their independent origins, however, many of the effector proteins possess tandem repeats of segments containing EPIYA-like motifs in their predicted IDRs [8]. Hence, IDRs are likely to be a critical feature of these effector proteins in performing their functions and evolving into potent multifunctional binding sites. CD spectroscopy analysis suggested the presence of IDRs in the enteropathogenic *E. coli* Tir [68]. However, crystal structures of these bacterial EPIYA effectors in their full-length forms are as yet unavailable, most probably because of the presence of IDRs.

Bacterial pathogens deliver effector proteins into host cells to perturb host-signaling pathways for immune evasion and establishment of infection. Thus, effector proteins often mimic host proteins because they undergo positive selection for molecules that effectively hijack host-signaling pathways and are advantageous in pathogen survival. If an IDR is critical for a host protein function that the bacterial effector protein mimics, then the bacterial effector may be selected for an IDR as well. This is most likely to be the case with the intrinsically disordered EPIYA-C segment of *H. pylori* CagA that mimics the IDRs of Gab proteins and EGFR that interact with SHP2, and MAP proteins, which are substrates for PAR1b [69,70,71]. Besides host function mimicry, employment of IDRs in effector proteins can have several advantages over structured regions. Firstly, with *H. pylori* CagA as an example, effector proteins can generate tandem repeats of target binding sites through repeat expansion by homologous recombination, which dramatically augments the binding affinities to its targets [46,47]. This would be an important feature since bacterial effector proteins need to generate higher binding affinities to their targets than cognate ligands to subvert host-signaling pathways. Secondly, repeat expansion followed by mutations would diversify the binding sequences, enabling pleiotropic effects through interaction with multiple host proteins. Thirdly, the flexibility of the IDR is key in creating cost-effective, high-density binding sites, which allow for simultaneous binding of multiple proteins. IDRs would also be less bulky than well-structured functional domains and would allow effector proteins to be compact enough to pass through the pili of the type III or type IV secretion system.

**Figure 3:**
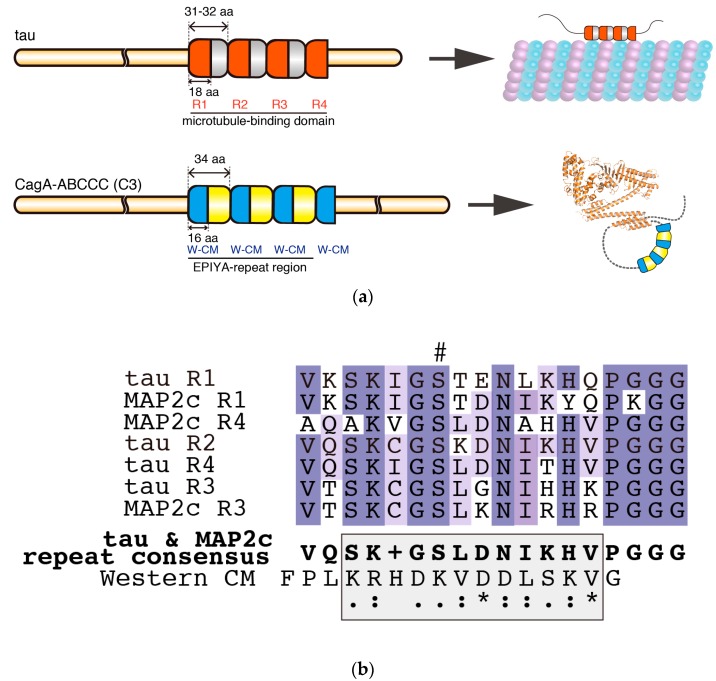
Comparison of the intrinsically disordered C-terminal repeat regions of tau and CagA. (**a**) The repeat regions of tau and CagA have similar segmental organizations. The 2N4R tau (RefSeq Accession: NP_005901.2) has a microtubule-binding domain that is comprised of four degenerate repeat segments. Each of these 31–32 amino-acid repeat segments contains a semi-conserved 18 amino-acid core repeat termed R1–R4. The microtubule-binding domain binds and stabilizes microtubules; however, tau itself remains intrinsically disordered. R1–R4 of tau are targets of PAR1b and their phosphorylation results in dissociation of tau from microtubules. In comparison, the EPIYA-repeat region of ABCCC-type CagA (NCTC11637 strain) is comprised of three highly conserved 34 amino-acid EPIYA-C segments, each of which contains a fully conserved 16 amino-acid Western CM motif. Another fully conserved Western CM motif also exists immediately distal to the third repeat of the EPIYA-C segments. The CM motifs are located in the C-terminal tail of CagA, which forms a flexible lariat loop; (**b**) Multiple sequence alignment of tau and MAP2c (RefSeq Accession: NP_114033-2) R1-R4 repeats revealed a consensus sequence that showed weak similarity (shaded box) with the Western CM sequence. Hash denotes phosphorylation target site of PAR1b. Multiple sequence alignment and consensus sequence were generated by Clustal Omega [78].

Intriguingly, the structural organization of the C-terminal tail of CagA (C2 or C3) closely resembles MAPs, which are phosphorylated by PAR1 (Figure 3a). MAPs are IDPs that contain a microtubule-binding domain at their C termini, which, depending on the isoform, contains 3–5 repeat segments of 31–32 amino acids [72,73]. Each repeat segment contains a core semi-conserved repeat of 18 residues called the microtubule-binding repeat, which contains a phosphorylation site targeted by PAR1 (Figure 3a,b) [50,74]. It has been suggested that MAPs require phosphorylation at multiple sites in their repeat region to dissociate rapidly from microtubules [75,76,77]. Moreover, evidence so far from NMR and electron microscopy has suggested that MAPs are intrinsically disordered even when they are bound to microtubules [71]. Thus, the tandem repeats may be optimally spaced for efficient phosphorylation by a single molecule of PAR1. These findings point to the possibility that CagA with multiple EPIYA-C segments, each of which contains a PAR1-binding CM motif, may not only mimic host substrates in terms of its sequence (Figure 3b) but may also mimic the structural organization of the repeat region of MAPs to efficiently capture, release, and recapture PAR1 at neighboring binding sites (Figure 3a). It is therefore possible that the EPIYA motif-containing region of CagA is a structural example of convergent evolution that gives rise to enhanced PAR1 binding. Moreover, formation of a lariat loop may confer some restriction on the dynamic movement of the C-terminal CagA tail, which may be analogous to the restricted movement of MAPs when they are bound to microtubules. Further biochemical and biophysical analyses are required to determine if there is such a structural analogy between CagA and MAPs in terms of PAR1 binding.

## 6. Conclusions and Perspectives

Sequence polymorphisms involving EPIYA and CM motifs play crucial roles in determining the magnitude of pathophysiological action of the *H. pylori* CagA oncoprotein. Quantitative analyses of *H. pylori* CagA polymorphisms showed that *H. pylori* has employed two strategies for increasing binding affinity to its targets SHP2 and PAR1b. Western CagA has acquired enhanced binding to SHP2 and PAR1b through repeat expansion of the EPIYA-C segment, which contains both the EPIYA and the CM motifs. East Asian CagA, which cannot facilitate homologous recombination since it only contains one *CM* sequence at the 3′ end of the region encoding the EPIYA-D segment, has instead accumulated mutations in the EPIYA-D segment and the CM motif to potentiate its binding affinity to SHP2 and PAR1b, respectively. Results of these quantitative studies offer a new perspective for understanding the molecular mechanisms underlying the relationship between sequence polymorphisms of CagA and risk of gastrointestinal diseases. Other bacterial pathogens also produce effector proteins that have sequence polymorphisms in IDRs [8]. IDRs are also abundant in the proteomes of viruses and pathogenic protozoans [79,80]. Thus, IDRs are a common feature of pathogenic proteins that perturb host-signaling pathways for establishment of infection. Recent studies on the sequence polymorphisms of CagA may shed light on how the sequence polymorphisms of other effector proteins affect their pathophysiological activities.

A recent study has suggested that *H. pylori* CagA could be transported via the exosomes from *H. pylori*-infected gastric epithelial cells to remote organs, which provides a novel perspective on how *H. pylori* can elicit extragastric diseases [81]. Indeed, epidemiological studies have shown that cardiovascular, hematological and neurological disorders are linked to infection with *cagA*-positive *H. pylori* [82,83,84,85]. Since only a small amount of CagA can be enclosed in exosomes that are then circulated to vast areas of the body, only minute amounts of CagA may be delivered to remote tissues. If this turns out to be the case, CagA with a larger number of EPIYA-C repeats may be more potent in provoking target tissue damage. It would therefore be interesting to see in the future if there is an epidemiological link between CagA polymorphisms and the risk of extragastric diseases.
